# Uric Acid Contributes to Obesity-Paradox of the Outcome of Ischemic Stroke

**DOI:** 10.3389/fneur.2019.01279

**Published:** 2019-12-05

**Authors:** Hefei Tang, Jinglin Mo, Zimo Chen, Jie Xu, Anxin Wang, Liye Dai, Aichun Cheng, Yongjun Wang

**Affiliations:** ^1^Department of Neurology, Beijing Tiantan Hospital, Capital Medical University, Beijing, China; ^2^China National Clinical Research Center for Neurological Diseases, Beijing, China; ^3^Center of Stroke, Beijing Institute for Brain Disorders, Beijing, China; ^4^Beijing Key Laboratory of Translational Medicine for Cerebrovascular Disease, Beijing, China

**Keywords:** uric acid, body mass index, ischemic stroke, obesity-paradox, clinical outcomes

## Abstract

**Background:** The mechanism of obesity paradox in stroke is not clear. This study aimed to investigate whether uric acid (UA) contributes to obesity-stroke outcome paradox.

**Material and Methods:** The study cohort consisted of 1,984 IS patients recruited in the ACROSS-China study. Serum UA and BMI were measured at admission. Low and high BMI groups were defined by the threshold of 24, and low and high UA by the age- and sex-specific median. Poor outcomes were defined as modified Rankin scale score ≥3 in 1 year after onset.

**Results:** UA was significantly and positively correlated with BMI. Lower levels of UA and BMI were significantly associated with higher risk of poor outcomes. Incidence of the poor outcome was 34.5, 29.4, 27.7, and 23.5% in the BMI/UA groups of low/low, high/low, low/high and high/high, respectively, with *p* = 0.001 for trend. The association between low UA and poor outcome was significant in lower BMI groups (odds ratio = 1.36, *p* = 0.006 in quartile 1 and 1.28, *p* = 0.021 in quartile 2), but the odds ratios were not significant in the BMI quartile 3 and 4 groups, with *p* = 0.038 for trend. The adverse effect of lower UA was significant in males, but not in females, with *p* = 0.006 for sex difference.

**Conclusions:** These findings suggest that low UA and low BMI have a joint effect on poor outcomes in IS patients. Across BMI categories, uric acid is differentially associated with functional outcome after stroke. This effect of low UA in the low BMI groups may be one of the mechanisms underlying the obesity-stroke paradox of the outcome in IS patients.

## Introduction

Uric acid is the final oxidation product of purine metabolism and a water-soluble antioxidant and radical scavenger in humans (1, 2). Despite the antioxidant properties of uric acid, epidemiologic studies have shown that hyperuricemia is related to an increased risk of cardiovascular events, stroke, and their risk factors (3–6). On the other hand, growing evidence has been emerging that elevated uric acid concentration is associated with better functional outcomes of ischemic stroke (IS) (7–11). Moreover, the exogenous administration of uric acid exerts robust neuroprotective effect on the outcome of IS patients in clinical trials (12, 13).

Obesity, a metabolic disorder, has a wide range of negative effects on health consequences (14). There is consistent evidence that obesity is an established independent risk factor for incidence of IS (15). However, many studies, but not all, suggest a better prognosis in overweight and obese patients after IS (16–19). The improved survival and functional outcomes, and stroke recurrence in overweight and obese IS patients are termed as “obesity-stroke paradox” in the literature (18, 19). Although the obesity-stroke paradox related factors and metabolic pathways have been documented in previous studies, the underpinning mechanisms are still not fully understood (20, 21).

Oxidative stress is a major contributor to brain damage in patients with IS (22). Uric acid, a powerful antioxidant, is highly and positively correlated with body weight (6). To date, the joint effect of uric acid and obesity on the IS outcomes has not been reported in previous studies. The working hypothesis of this study is that uric acid concentration contributes to the obesity-stroke outcome paradox in patients with IS. The current study aims to investigate the synergistic effect of uric acid and body mass index (BMI) on clinical functional outcomes after IS in a 1-year follow-up study cohort.

## Materials and Methods

### Participants and Procedures

The Abnormal gluCose Regulation in patients with acute strOke acroSS China (ACROSS-China) is a hospital-based multicenter prospective cohort study. The ACROSS-China study focuses on the prevalence and distribution of abnormal glucose regulation in patients with acute stroke and the impact of abnormal glucose regulation on the outcome of stroke patients (11). A total of 3,450 patients were recruited from 34 hospitals across China in 2008–2009, including patients with IS (*n* = 2,639), intracerebral hemorrhage (*n* = 649) and subarachnoid hemorrhage (*n* = 162). Acute stroke was diagnosed according to World Health Organization diagnostic criteria (23). The patients were followed for 1 year, and information on clinical outcomes was obtained by personal interview. Among the 2,639 patients with IS, 1,984 patients who had serum uric acid data available and completed the follow-up interview survey for the outcome formed the current study cohort. This study is a retrospective analysis of the ACROSS-China cohort. Characteristics of the patients who were included and excluded (*n* = 655) were presented in Supplemental Table 1. The study was approved by the Central Institutional Review Board at Beijing Tiantan Hospital. Written informed consent was obtained from all the patients or their representatives.

Replicate measurements of height and weight were made, and the mean values were used for analysis. BMI (weight in kilograms divided by height in meters squared) was used to measure adiposity. Low and high BMI was defined by the threshold of 24 kg/m2, based on recommendations of the Working Group on Obesity in China (24). Serum uric acid was measured at admission by the urine enzyme endpoint method. Low and high uric acid was defined as being below and above the age- and sex-specific medians. Fast glucose was measured by using an enzymatic method. Estimated glomerular filtration rate (eGFR) was calculated using the Chronic Kidney Disease Epidemiology Collaboration creatinine equation with adjusted coefficient of 1.1 for the Asian population. Information, including age, sex, smoking, alcohol drinking, disease history, time from symptom onset to admission and medications was obtained by means of a nurse-administered standardized questionnaire at the time of admission. Current smoking and drinking were defined as smoking at least one cigarette per day and consuming alcohol every day, respectively.

At 1 year after stroke onset, the prognosis of all the patients were assessed through a centralized telephone follow-up. The primary clinical outcome of this study was defined as death or major disability at the end of 1-year follow-up period. Modified Rankin scale (mRS) score ranging from 3 to 6 was defined as major disability or death. A score of 0 indicated no symptoms, a score of 5 indicated severe disability, i.e., bedridden, incontinent, or requiring constant nursing care and attention, and a score of 6 indicated death (25). Death events were confirmed through death certificates from the local citizen registry or by the attended hospital.

### Statistical Analyses

Mean ± standard deviation (SD) or median (interquartile range [IQR]) was used for describing continuous variables, and frequencies and percentages for categorical variables. Differences in distribution of dichotomous variables between subgroups were tested by Chi-square tests. Uric acid and BMI were analyzed as both continuous and dichotomous variables. The relationship of BMI and uric acid with the outcome was examined in multivariable logistic regression models. Covariates included in the model for adjustment were age, sex, time from symptom onset to admission, smoking, alcohol drinking, National Institutes of Health stroke scale (NIHSS), stroke subtype, fast glucose, eGFR, blood pressure, and lipid-lowering and antihypertensive medications. Odds ratio (OR) and 95% confidence interval (CI) were estimated in logistic regression models. The significance of differences in ORs of uric acid between BMI quartile groups was tested in logistic regression interaction models. All statistical analyses were performed using SAS software (version 9.4; SAS Institute Inc., Cary, NC, USA).

## Results

Table 1 summarizes characteristics of study variables by sex groups and in the total sample. Males were younger and had greater prevalence of smokers and alcohol drinkers, lower prevalence of anti-hypertensive treatment and lower glucose, NIHSS than females. Males had lower BMI but higher UA and eGFR than females. The difference in incidence rates of poor outcome (mRS ≥ 3) between sex groups was significant, with males having a lower rate than females.

**Table 1:** Characteristics of ischemic stroke patients by sex groups.

**Variable**	**Total (*n* = 1984)**	**Males (*n* = 1263)**	**Females (*n* = 721)**	*P*-value^*^
Age (year)	62.4 (12.5)	60.5 (12.6)	65.6 (11.7)	<0.001
Smokers, *n* (%)	720 (36.3)	680 (53.8)	40 (5.6)	<0.001
Drinkers, *n* (%)	774 (39.0)	741 (58.7)	33 (4.6)	<0.001
Anti-hypertensive medication	875 (44.1)	518 (41.0)	357 (49.5)	<0.001
Lipid-lowering medication	1437 (72.4)	927 (73.4)	510 (70.7)	0.202
NIHSS, median, (IQR)	2 (1–5)	2 (1–5)	2 (1–6)	0.018
TOAST subtype, *n* (%)				<0.001
• Large-artery atherosclerosis	1257 (63.4)	803 (63.6)	454 (63.0)	
• Small-artery occlusion	494 (24.9)	336 (26.6)	158 (21.9)	
• Cardioembolism	118 (6.0)	46 (3.6)	72 (10.0)	
• Other	115 (5.8)	78 (6.2)	37 (5.1)	
Systolic BP, mean (SD), (mm Hg)	146.6 (19.6)	145.9 (19.5)	147.9 (19.8)	0.057
Diabetes, *n* (%)	435 (21.9)	267 (21.1)	168 (23.3)	0.263
Glucose, mean (SD), (mmol/L)	6.6 (2.8)	6.5 (2.7)	6.9 (3.0)	<0.001
eGFR, median, (IQR), ml/min/1.73 m2	98.1 (81.3–111.2)	100.2 (83.4–112.7)	95.0 (78.4–108.1)	<0.001
BMI, mean (SD), (kg/m^2^)	24.9 (3.8)	24.8 (3.3)	25.2 (4.5)	0.002
Uric acid, mean (SD), (μmol/L)	302.2 (98.9)	319.1 (97.8)	272.6 (93.9)	<0.001
Time from symptom onset to admission, median, (IQR), h	2 (1–5)	2 (1–5)	2 (1–5)	0.574
One-year outcomes				
• mRS median, (IQR)	1 (1–3)	1 (0–2)	1 (1–3)	<0.001
• mRS≥3, *n* (%)	562 (28.3)	315 (24.9)	247 (34.3)	<0.001
• Death, *n* (%)	252 (12.7)	153 (12.1)	99 (13.7)	0.298

*BP, blood pressure; NIHSS, National Institutes of Health stroke scale; TOAST, Trial of Org 10172 in Acute Stroke Treatment; BMI, body mass index; mRS, modified Rankin Scale; SD, standard deviation*.

* *P-values for difference between sex groups*.

Table 2 shows the relationship of uric acid, BMI, age, sex, and diabetes. Female sex was significantly associated with lower uric acid, but higher BMI. Older age was significantly associated with higher uric acid and lower BMI. IS patient with diabetes had lower uric acid, but higher BMI. Of note, BMI was significantly and positively correlated with uric acid.

**Table 2:** Relationship between uric acid, BMI, age, sex, and diabetes.

	**Dependent Variable: Uric Acid**		**Dependent Variable: BMI**	
	**β (SE)**	***p***	**β (SE)**	***p***
Female sex	−49.3 (4.6)	<0.001	0.58 (0.18)	0.001
Age	0.50 (0.18)	0.005	−0.04 (0.01)	<0.001
Diabetes	−23.0 (5.2)	<0.001	0.56 (0.20)	0.006
BMI	2.0 (0.6)	<0.001	—	—

*BMI, body mass index; SE, standard error*.

**Figure 1:**
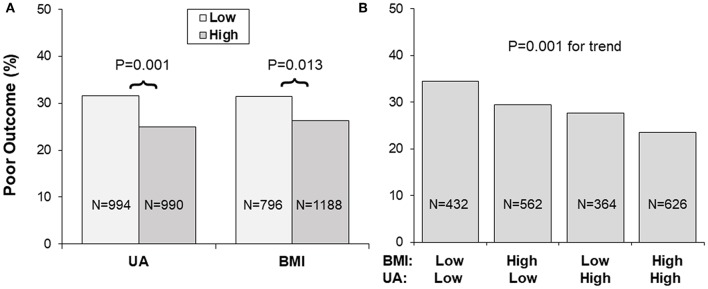
**(A,B)** Incidence of poor outcome (mRS ≥ 3) by BMI and uric acid groups. Low and high BMI was defined by the threshold of 24, and low and high UA by the sex-specific medians. BMI, body mass index; mRS, modified Rankin Scale; UA, uric acid.

Figure 1 presents incidence rates of poor outcome (mRS ≥ 3) in 1-year follow-up by uric acid and BMI groups. Low and high BMI was defined by the threshold of 24, and low and high uric acid by the median. As shown in **Panel A**, the lower uric acid group showed a higher incidence rate of poor outcome than the higher uric acid group (31.6 vs. 25.0%, *p* = 0.001); the lower BMI group showed a higher incidence rate of poor outcome than the higher BMI group (31.4 vs. 26.3%, *p* = 0.013). **Panel B** showed incidence rates of poor outcome by groups of combined BMI/uric acid. Incidence of the poor outcome was 34.5, 29.4, 27.7, and 23.5% in the BMI/uric acid groups of low/low, high/low, low/high and high/high, respectively, with *p* = 0.001 for trend.

Table 3 shows the association of BMI and uric acid with outcome, adjusting for covariates. Older age was significantly associated with higher risk of worse outcome. Patients with higher NIHSS were 1.26 times more likely to have worse outcome. Higher blood glucose was significantly associated with higher risk of poor outcome. Higher levels of BMI and uric acid had significantly protective effects on the outcome. The interactions of uric acid were significant with sex (*p* = 0.001) and BMI (*p* = 0.045), but not significant with age (*p* = 0.313), systolic blood pressure (*p* = 0.487) and diabetes (*p* = 0.974).

**Table 3:** Odds ratio (OR) of uric acid for poor outcome (mRS ≥ 3), adjusting for covariates.

	**OR**	**95% CI**	***P***
Uric acid, mean (SD), (μmol/L)	0.999	0.997–1.000	0.029
Age, (year)	1.048	1.037–1.059	<0.001
Sex, (male)	0.944	0.703–1.268	0.703
Smoking, *n* (%)	0.919	0.684–1.235	0.574
Alcohol drinking, *n* (%)	1.036	0.769–1.395	0.816
Anti-hypertensive medication, *n* (%)	1.043	0.818–1.330	0.733
Lipid-lowering medication, *n* (%)	1.027	0.792–1.331	0.842
NIHSS, median, (IQR)	1.257	1.218–1.298	<0.001
TOAST subtype			
• Large-artery atherosclerosis	0.527	0.280–0.993	0.009
• Small-artery occlusion	0.959	0.725–1.267	0.921
• Cardioembolism	1.749	1.040–2.944	0.002
• Other	Reference	Reference	
Systolic blood pressure, (mmHg)	0.999	0.993–1.005	0.755
Diabetes, *n* (%)	1.104	0.821–1.485	0.513
BMI, mean (SD), (kg/m^2^)	0.963	0.933–0.995	0.022
Time from symptom onset to admission, median, (IQR), h	0.902	0.715–1.138	0.384
Glucose mean (SD), (mmol/L)	2.084	1.602–2.710	<0.001
eGFR, median, (IQR), ml/min/1.73 m^2^	1.538	1.000–2.366	0.050

*NIHSS, National Institutes of Health stroke scale; TOAST, Trial of Org 10172 in Acute Stroke Treatment; BMI, body mass index; mRS, modified Rankin Scale; CI, confidence interval; OR, Odds ratio*.

**Figure 2:**
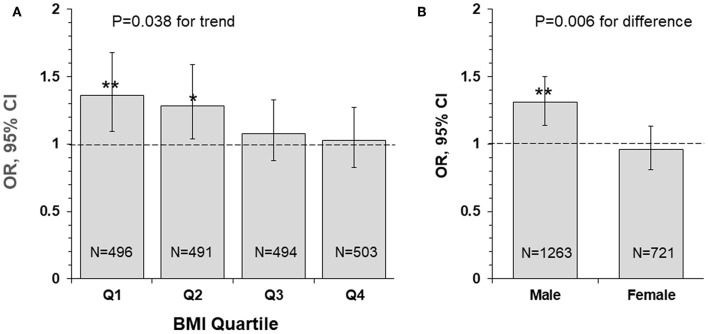
Odds ratio and 95% confidence interval of lower uric acid for poor outcome (mRS ≥ 3) by BMI quartile **(A)** and sex **(B)** groups, with quartile 4 of uric acid (the highest) as reference. *P < 0.05 and **P < 0.01 for OR >1. BMI, body mass index; CI, confidence interval; mRS, modified Rankin Scale; OR, Odds ratio.

Figure 2 presents odds ratio (OR) and 95% confidence interval (CI) of lower uric acid for poor outcome (mRS ≥ 3) by BMI quartile (panel A) and sex (panel B) groups, with quartile 4 of uric acid (the highest) as reference. The association between lower uric acid and poor outcome was significant in lower BMI groups (OR = 1.36, *p* = 0.006 in quartile 1; OR = 1.28, *p* = 0.021 in quartile 2), but the ORs were not significant in BMI quartile 3 and 4 groups. The interaction between uric acid and BMI was significant, with *p* = 0.038 for the decreasing trend in ORs across increasing BMI quartiles. The association between low uric acid and poor outcome was significant in males (OR = 1.31, *p* < 0.001), but not significant in females (OR = 0.96, *p* = 0.618), with *p* = 0.006 for sex difference.

**Figure 3:**
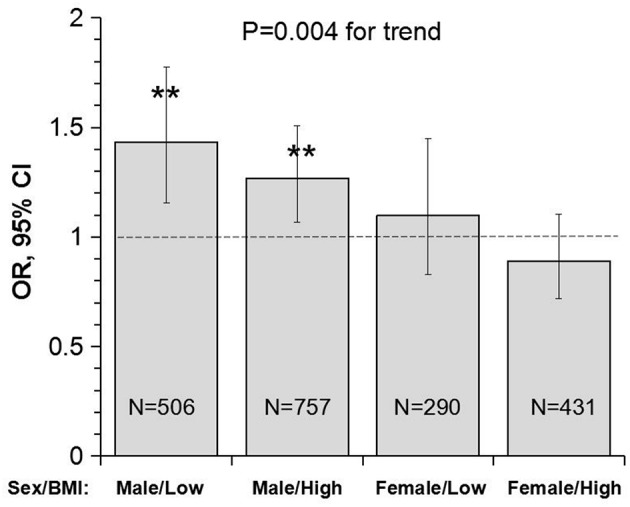
Odds ratio and 95% confidence interval of lower uric acid for poor outcome (mRS ≥ 3) with quartile 4 of uric acid (the highest) as reference by sex/BMI groups. Low and high BMI was defined by the threshold of 24; **P < 0.01 for OR >1. BMI, body mass index; CI, confidence interval; mRS, modified Rankin scale; OR, Odds ratio.

Figure 3 presents OR and 95% CI of lower uric acid for poor outcome by sex/BMI groups, with quartile 4 of uric acid (the highest) as reference. The adverse effect of lower uric acid measured as OR was significant in males, but not in females, with *p* = 0.004 for the trend. Of note, the difference in ORs between low and high BMI groups was not significant in males (*p* = 0.512 for interaction) and females (*p* = 0.335 for interaction).

## Discussion

Obesity is an escalating pandemic worldwide, representing an emerging threat to public health (26, 27). The obesity association with cardiovascular and cerebrovascular diseases is well-demonstrated. Studies have concluded that obesity is strongly associated with increased stroke risk in both men and women (28, 29). Although the adverse health consequences of obesity in the general population have been well-documented, strong evidence suggests that obesity is associated with improved survival rate and functional outcomes and lower stroke recurrence in patients with IS, indicating the existence of an “obesity-stroke paradox” (16–19). However, some studies supported that no obesity paradox exists in patients with IS after adjustment for initial neurological severity (30–32). The existence of obesity paradox is still controversial. In the current study, the higher BMI (≥24) group had a significantly lower incidence of poor functional outcome (mRS ≥ 3) than the lower BMI (<24) group (26.3 vs. 31.4%, *p* = 0.013) in a 1-year follow-up of IS patients; BMI was significantly and negatively associated with the risk of poor functional outcome in multivariable logistic regression models, adjusting for covariates. Despite strong observational data indicating a survival benefit of obese patients after stroke, methodological concerns exist (16, 33), and the biologic mechanisms contributing to pathways in metabolic imbalance underlying the obesity-stroke paradox are largely unknown (20, 21).

Data from most observational studies, systematic reviews, and meta-analyses support the concept that elevated uric acid concentration is associated with better functional outcomes of IS (7–11). Patients with acute IS have a 12% increase in the odds of good clinical outcome for each milligram per deciliter increase of serum uric acid (34). Moreover, a meta-analysis of 10 studies found that high serum uric acid level was associated better outcome after acute IS as compared to low serum uric acid level (9). Furthermore, one prospective study suggested that the increased uric acid levels are associated with better outcome in IS patients treated with reperfusion therapy (35). Similarly, another study demonstrated that early elevation of uric acid during or shortly after IS onset presented significant protection against neurological deficit in acute IS patients treated with recombinant tissue plasminogen activator (rt-PA) (36). The ACROSS-China study reported that lower serum uric acid levels strongly predicted short-term poor functional outcome in acute stroke with normoglycaemia (11). In addition, evidence for the beneficial effect of exogenous administration of uric acid on the outcome of IS patients in clinical trials has been emerging in recent years (12, 13). We found in this study that lower uric acid levels were significantly associated with poor functional outcome of IS patients, and this effect was exacerbated by lower BMI. The joint effect of uric acid and obesity status on the IS outcome has not been reported in previous studies. The detrimental impact of lower uric acid observed in the present study may be one of metabolic mechanisms of the obesity-stroke outcome paradox.

Obesity, a metabolic disorder, is well-known to correlate with uric acid. The Bogalusa Herat Study has shown that BMI levels were significantly, positively associated with uric acid in both children and adults (6). It is also noted in current study cohort that lower BMI was significantly correlated with decreased levels of uric acid in IS patients. Incidence of poor outcome (mRS ≥ 3) was the highest in the low BMI-low uric acid group and was the lowest in the high BMI-high uric acid group, with p for trend = 0.001 (Figure 1B). Uric acid concentrations decrease significantly over time in stroke patients, and the plasma antioxidant capacity has been inversely correlated with the volume of cerebral infarction and the severity of neurological impairment (37). IS patients with lower BMI tended to have lower uric acid which was associated with decreased antioxidant capacity. The highest OR of low uric acid for poor outcome in IS in the lowest BMI quartile group (Figure 2A) indicated a synergistic effect of lower BMI and lower uric acid on the poor outcome. The findings on the interaction effect between BMI and uric acid on the IS outcome suggested that lower BMI levels might be associated with poor outcome in IS, at least in part, through lower uric acid concentrations. Studies with specifically sophisticated design in other populations are needed to confirm our findings. Further investigations should be performed in acute IS patient treated with rt-PA to further explore whether same results can be observed.

Another interesting finding in this study is the sex-specific association between uric acid and the outcome in IS. Despite the significant difference in the incidence of poor outcome (males < females), the association between lower uric acid and the poor outcome was significant in male patients, but not in female patients. The male-low BMI group showed the highest OR (1.43), and the female-high BMI group showed the lowest OR (0.89), with p for trend = 0.004 (Figure 3B). In a prospective study of the China Antihypertensive Trial in Acute Ischemic Stroke, the association between serum uric acid and primary outcomes of IS measured as death and major disability (mRS ≥ 3) at 3 months was modified by sex (P for interaction *n* = 0.007) in 3284 acute IS patients. Elevated serum uric acid was significantly and positively associated with the primary outcome in men, but not in women (38). In contrast, the administration of uric acid reduced infarct growth in patients with acute IS treated with alteplase in women, but not in men in a clinical trial in Spain (39). As we know, uric acid level is different between sex, and men usually have a higher uric acid level than women. Women with higher uric acid levels were significantly associated with the development of hypertension and metabolic syndrome, however, it was not found in men. We hypothesized that exogenous administration of uric acid may have a greater benefit for male patients with low uric acid level in acute ischemic stroke with or without reperfusion therapies. To date, data on the sex-specific association between uric acid and outcomes in IS are still limited. Prospective double blind randomized controlled trials in this regard are required to investigate the effect of uric acid on the outcomes in male and female IS patients.

There are several limitations of our study include: First, the result of this study could not generalized to western population, because there is much difference between western population and Chinese population in the distribution of BMI. Second, our study has significant missing data about serum uric acid and the included population has higher proportion of diabetes, lipid-lowering medication, and higher BMI level compared with excluded population. Thus, popularizing the results to general population is limited. Further investigations in other larger populations are needed to confirm the findings. Third, the telephone follow-up for outcome assessment is sub-optimal. Fourth, this study is a hospital-based cohort, which may lead to selection bias. A large population-based study is needed to confirm our findings. Fifth, the cohort was recruited between 2008 and 2009, therefore, it was considered not contemporary. Sixth, the lack of information on use of acute reperfusion therapies in this study may hampered further investigation on the relationship between uric acid and obesity-stroke paradox in patients treated with reperfusion therapies.

## Conclusions

In summary, this observational prospective study highlighted that across BMI categories, uric acid was differentially associated with functional outcome after stroke. Of note, a synergistic effect of uric acid and BMI was found that the association between low levels of uric acid and the poor clinical functional outcome was strengthened by lower BMI in IS patients. The detrimental effect of low uric acid on the outcome was observed to be significant in male, but not in female IS patients. The findings of the present study have implications in understanding the metabolic mechanisms of the obesity-stroke outcome paradox, help identify high-risk patients of IS and provide additional evidence for the administration of uric acid in clinical trial studies. Given the practical implications that could be derived in the field of prevention of poor outcome in IS, larger studies would be required to confirm these encouraging results.

## Data Availability Statement

The datasets generated for this study are available on request to the corresponding author.

## Author Contributions

YW had full access to all of the data in the study and takes responsibility for the integrity of the data and the accuracy of the data analysis. Study concept and design were done by HT, JM, and ZC. Acquisition, analysis, or interpretation of data were done by LD, AC, and JX. Drafting of the manuscript was done by HT, JM, and ZC. Statistical analysis was done by AW. Study supervision was the responsibility of YW.

### Conflict of Interest

The authors declare that the research was conducted in the absence of any commercial or financial relationships that could be construed as a potential conflict of interest.
