# Supplementary material for: Uric Acid Contributes to Obesity-Paradox of the Outcome of Ischemic Stroke
Source: Front Neurol. 2019 Dec 5;10:1279. doi: 10.3389/fneur.2019.01279 (PMC6906190; doi:10.3389/fneur.2019.01279)
Supplement: Supplementary file 1 [file Table_1.DOC]

**Table e-1.** Characteristics of ischemic stroke patients included and excluded

| Variable | Included  (n=1984) | Excluded  (n=655) | P value* |
| --- | --- | --- | --- |
| Age (year) | 62.4 (12.5) | 63.8 (12.5) | 0.013 |
| Males n (%) | 1263 (63.7) | 419 (65.1) | 0.519 |
| Smokers, n (%) | 720 (36.3) | 231 (35.3) | 0.636 |
| Drinkers, n (%) | 774 (39.0) | 246 (37.6) | 0.507 |
| Anti-hypertensive medication n (%) | 875 (44.1) | 287 (43.8) | 0.898 |
| Lipid-lowering medication n (%) | 1437 (72.4) | 443 (67.6) | 0.019 |
| NIHSS, mean (SD) | 3.7 (4.4) | 4.1 (4.6) | 0.064 |
| TOAST subtype, n (%) |  |  | 0.892 |
| Large-artery atherosclerosis | 1257 (63.4) | 393 (60.0) |  |
| Small-artery occlusion | 494 (24.9) | 165 (25.2) |  |
| Cardioembolism | 118 (5.9) | 43 (6.6) |  |
| Other | 115 (5.8) | 54 (8.2) |  |
| Systolic BP, mean (SD), (mm Hg) | 146.6 (19.6) | 146.0 (20.2) | 0.389 |
| Diabetes, n (%) | 435 (21.9) | 99 (15.1) | <0.001 |
| BMI, mean (SD), (kg/m^2^) | 24.9 (3.8) | 24.4 (3.5) | 0.003 |
| One-year outcomes† |  |  |  |
| mRS, mean (SD) | 1.9 (2.0) | 2.0 (2.0) | 0.945 |
| mRS≥3, n (%) | 562 (28.3) | 44 (32.6) | 0.289 |
| Death, n (%) | 252 (12.7) | 15 (11.1) | 0.590 |

**Abbreviations:** BP, blood pressure; NIHSS, National Institutes of Health stroke scale; TOAST, Trial of Org 10172 in Acute Stroke Treatment; BMI, body mass index; mRS, modified Rankin Scale; SD, standard deviation.

**Note:** * P-values for difference between sex groups;

† Among participants who were excluded, there were 135 patients who had mRS score.
